# Revision ACL reconstruction, but not bilateral ACL reconstruction, is associated with clinically relevant inferior subjective knee function compared with primary ACL reconstruction: A comparative analysis of 6831 patients

**DOI:** 10.1002/ksa.12496

**Published:** 2024-10-03

**Authors:** Riccardo Cristiani, Eric Hamrin Senorski, Camilo P. Helito, Kristian Samuelsson, Anders Stålman

**Affiliations:** ^1^ Department of Molecular Medicine and Surgery Section of Sports Medicine, Karolinska Institutet Stockholm Sweden; ^2^ Stockholm Sports Trauma Research Center (SSTRC), FIFA Medical Centre of Excellence Stockholm Sweden; ^3^ Department of Orthopaedics, Institute of Clinical Sciences, The Sahlgrenska Academy University of Gothenburg Gothenburg Sweden; ^4^ Grupo de Joelho, Instituto de Ortopedia e Traumatologia, Hospital das Clínicas HCFMUSP, Faculdade de Medicina da Universidade de São Paulo São Paulo Brazil; ^5^ Hospital Sírio Libanês São Paulo Brazil

**Keywords:** ACL, anterior cruciate ligament, bilateral ACL reconstruction, KOOS, revision ACL reconstruction, subjective knee outcome

## Abstract

**Purpose:**

To evaluate and compare the subjective knee function in patients undergoing revision and bilateral anterior cruciate ligament (ACL) reconstruction (ACLR) with those undergoing primary ACLR in a large cohort.

**Methods:**

Patients without concomitant ligament injuries who underwent primary, revision or bilateral ACLR at the Capio Artro Clinic, Stockholm, Sweden, between 2005 and 2018 were identified. The Knee injury and Osteoarthritis Outcome Score (KOOS) was collected preoperatively and at 1, 2 and 5 years postoperatively from the Swedish National Knee Ligament Registry. Patients who underwent revision and bilateral ACLR were compared with those who underwent primary ACLR (control group) using Student's *t* test.

**Results:**

A total of 6831 patients (6102 with primary ACLRs, 343 with revision ACLRs and 386 with bilateral ACLRs) were included. Preoperatively, there were significant but nonclinically relevant differences in favour of the revision ACLR group for KOOS Symptoms, Pain, Activities of Daily Living (ADL) and Sport/Rec subscale scores compared with the primary ACLR group. Postoperatively, except for the 1‐year Symptoms and ADL subscales, the revision ACLR group reported significantly lower scores on all KOOS subscales than the primary ACLR group, with clinically relevant differences (>8–10 points) for the 1‐, 2‐ and 5‐year Sport/Rec and Quality of Life (QOL) subscales. The bilateral ACLR group reported significantly, but not clinically relevant, inferior scores on the 1‐year Symptoms and QOL subscales and the 5‐year Sport/Rec and QOL subscales compared with the primary ACLR group.

**Conclusions:**

Revision ACLR, but not bilateral ACLR, was associated with clinically relevant inferior subjective knee function compared with primary ACLR. It is important to counsel patients regarding their future subjective knee function after repeated ACLR. Compared to primary ACLR, inferior subjective results should be expected after revision ACLR, but not after bilateral ACLR.

**Level of Evidence:**

Level III.

**Abbreviations**
ACL: anterior cruciate ligament
ACLR: anterior cruciate ligament reconstruction
ADL: Activities of Daily Living
BPTB: bone patellar tendon bone
HT: hamstring tendon
KOOS: Knee Injury and Osteoarthritis Outcome Score
QOL: Quality of Life
QT: quadriceps tendon
SNKLR: Swedish National Knee Ligament Registry

## INTRODUCTION

The incidence of anterior cruciate ligament (ACL) reconstruction (ACLR) has recently increased [24, 28, 39]. The risk of sustaining a new ACL injury in either knee is much higher in patients with an ACL‐reconstructed knee than in those with healthy knees [13]. It has been reported that 23%–25% of young athletes who underwent ACLR and returned to pivoting sports sustained a new ACL tear, with 55%–75% occurring in the contralateral knee [15, 27]. Paterno et al. [27] reported that the incidence rate of ACL injury in the same knee during the first postoperative year (in athletes aged between 10 and 25 years) after ACLR was 15 times greater than that in healthy knee controls.

The literature is scarce and inconsistent regarding comparisons of subjective knee function between patients who underwent bilateral and unilateral ACLR. Koca et al. [21] reported inferior subjective knee function in patients who underwent bilateral ACLR compared to patients who underwent unilateral ACLR. In contrast, Goddard et al. [17] found no differences in subjective knee function between patients who underwent bilateral or unilateral ACLR. Similarly, Cristiani et al. [10] found that subjective knee function after contralateral ACLR was comparable to that after primary ACLR.

Several authors [1, 3, 16, 20, 37] have reported that patients who underwent revision ACLR have inferior subjective knee function compared with patients who underwent primary ACLR. However, Tomihara et al. [35] found that revision bone‐patellar tendon‐bone (BPTB) ACLR resulted in subjective knee function similar to that of primary BPTB ACLR. Similarly, a recent matched‐pair analysis with 3–5 years of follow‐up found that revision ACLR results in similar subjective knee function as primary ACLR [25]. A clinical review [19] concluded that most studies comparing subjective knee function between patients with primary and revision ACLR had small sample sizes and short follow‐ups.

Patients undergoing revision or bilateral ACLR require thorough counselling regarding their expected subjective knee function postoperatively. Patient expectations after ACL surgery are often high and sometimes unrealistic, which has been shown to correlate negatively with patient‐reported outcomes postoperatively [14]. A large cohort study comparing subjective knee function between patients with revision or bilateral ACLR and patients with primary ACLR would be helpful in clinical practice to gain a clear understanding of the impact of the second (revision or contralateral) ACLR on subjective knee function and help surgeons to properly counsel patients about their expected outcomes after their second ACL surgery.

The purpose of this study was to evaluate and compare subjective knee function between revision or bilateral ACLR and primary ACLR in a large cohort. It was hypothesised that revision and bilateral ACLR would be associated with clinically relevant inferior subjective knee function compared to primary ACLR.

## MATERIALS AND METHODS

Ethical approval for this study was obtained from the regional ethics committee, Karolinska Institutet, Diarienumber 2016/1613‐31/32.

The Strengthening the Reporting of Observational Studies in Epidemiology statement was used as guidance to report this study to increase scientific quality [36].

Patients without others concomitant ligament injuries who underwent primary, revision or bilateral ACLR at Capio Artro Clinic, Stockholm, Sweden, from 2005 to 2018, were identified in our local database. All bilateral ACLRs were performed in two stages (primary ACLR and subsequent contralateral ACLR), whereas all revision ACLRs were performed as single stage.

### Surgical technique and rehabilitation

All ACLRs were performed by using a single‐bundle technique and an anteromedial portal technique to drill the femoral tunnel. For hamstring tendon (HT) ACLRs, the semitendinosus tendon was primarily harvested and prepared as a tripled or quadrupled graft. If the diameter was considered insufficient (<8 mm), the gracilis tendon was also harvested and combined with the semitendinosus tendon. For BPTB ACLRs, the central third of the patellar tendon with two bone blocks (approximately 20 mm) was harvested. The diameter of the BPTB graft was 9 or 10 mm. The defect in the central third of the patellar tendon was left open, whereas the paratendon was sutured with absorbable sutures. Quadriceps tendon (QT) grafts were harvested as full‐thickness tendon grafts from the central part of the QT with a bone block (approximately 20 mm) from the proximal pole of the patella. The diameter of the QT graft was 9 or 10 mm. All grafts were routinely fixed with an Endobutton (Smith & Nephew) or Tightrope (Arthrex) fixation device on the femoral side and Ethibond no. 2 sutures (Ethicon) tied over an AO bicortical screw with a washer as a post or with an interference screw on the tibial side. No additional procedures, such as lateral extraarticular tenodesis or anterolateral ligament reconstruction, were performed in any ACLR. During revision ACLR, removal of previous hardware was performed only if necessary (i.e., compromission of graft fixation or drilling of new tunnels). Meniscal repair was performed using an all‐inside technique with Fast‐Fix suture anchor devices (Smith & Nephew) for tears located in the posterior horn and body and with an outside‐in technique using PDS 0 (Ethicon) for tears located in the anterior horn of the meniscus. Rehabilitation was supervised and standardised. In cases of isolated ACLR or ACLR and meniscal resection, full weight‐bearing and range of motion were allowed, whereas in cases of ACLR and meniscal repair, the patients wore a hinged knee brace for 6 weeks (0–30° first and second week; 0–60° third and fourth week; 0–90° fifth and sixth week). From the seventh week, the brace was discontinued, and full range of motion was allowed. However, patients were recommended to avoid deep knee squatting for 3–4 months after meniscal repair. During the first 3 months, quadriceps strengthening was restricted to closed kinetic chain exercises for all patients. Return to sport was allowed at the earliest 6 months after surgery if isokinetic muscle strength and single‐leg‐hop test were satisfactory (limb symmetry index ≥90%) [6, 7, 34].

### Subjective knee function

The Knee Injury and Osteoarthritis Outcome Score (KOOS) [29, 30] was used to evaluate subjective knee function. The KOOS subscale scores were collected preoperatively and at the 1‐, 2‐ and 5‐year follow‐up. For the revision and bilateral ACLR groups, preoperative and postoperative (1, 2 and 5 years) scores were collected before and after the last surgery (revision and contralateral ACLR, respectively). The KOOS is divided in five subscales: Symptoms, Pain, Activities of Daily Living (ADL); Sports and Recreation (Sports/Rec) and knee‐related Quality of Life (QOL). A score of 0 is the worst possible outcome, whereas a score of 100 is the maximum score for each subscale. Differences of 8–10 points are considered to be clinically relevant [29, 31].

### Data sources

Age at surgery, sex, graft choice, meniscus surgery (resection or repair), cartilage injury and time intervals (from injury to primary ACLR, from primary to revision ACLR, and from primary to contralateral ACLR) were collected from our database. The KOOS subscale scores were extracted from the Swedish National Knee Ligament Registry [33].

### Statistical analysis

The statistical analysis was performed using the SPSS software (version 25.0; IBM Corp.). Standard descriptive statistics (frequency, mean and standard deviation) was used to summarise all variables. The distributions were checked for deviations from a normal distribution. Preoperative and postoperative KOOS subscale scores of the revision and bilateral ACLR groups were compared with those of the primary ACLR group (reference group) with Student's *t* tests. The significance level in all analysis was 5% (two‐tailed).

## RESULTS

A total of 6831 patients (6102 primary ACLRs, 343 revision ACLRs and 386 bilateral ACLRs) were included in the study. Patient characteristics are summarised in Table 1. The mean age at the time of primary ACLR was lower in the revision and bilateral ACLR groups, whereas the proportion of females was higher in the bilateral ACLR group. The most common graft at primary (for all groups) and contralateral ACLR (for the bilateral ACLR group) was the HT graft, whereas the most common graft at revision ACLR was BPTB. The cumulative prevalences of medial and lateral meniscal resections and cartilage injuries were higher in the revision ACLR group than in the primary ACLR group. Finally, the time from injury to primary ACLR was shorter in the revision and bilateral ACLR groups than in the isolated primary ACLR group (Table 1).

**Table 1:** Patient characteristics.

		Revision ACLR group		Bilateral ACLR group	
	Isolated primary ACLR	Primary ACLR	Revision ACLR	Primary ACLR	Contralateral ACLR
Patients	6102	343		386	
Age at surgery, mean ± SD, years	29.2 ± 10.9	23.1 ± 7.8	26.9 ± 8.9	24.5 ± 9.1	30.3 ± 10.4
Sex					
Male	3333 (54.6)	189 (55.1)		189 (49.0)	
Female	2769 (45.4)	154 (44.9)		197 (51.0)	
Graft					
HTs	5621 (92.1)	293 (85.4)	72 (21.0)	285 (73.8)	338 (87.6)
BPTB	349 (5.7)	49 (14.3)	255 (74.3)	100 (25.9)	45 (11.6)
QT	132 (2.2)	1 (0.3)	16 (4.7)	1 (0.3)	3 (0.8)
MM resection	919 (15.1)	39 (11.4)	45 (13.1)^a^	35 (9.1)	52 (13.5)
LM resection	954 (15.6)	54 (15.7)	37 (10.8)^b^	38 (9.8)	67 (17.4)
MM repair	500 (8.2)	18 (5.2)	30 (8.7)	13 (3.4)	21 (5.4)
LM repair	290 (4.8)	17 (5.0)	13 (3.8)	11 (2.8)	11 (2.8)
Cartilage injury	1126 (18.5)	33 (9.6)	69 (20.1)^c^	40 (10.4)	69 (17.9)
Time intervals, mean ± SD, months					
From injury to primary ACLR	21.2 ± 41.9	9.3 ± 16.7		13.7 ± 30.3	
Patients with available data	6029 (98.8)	282 (82.2)		252 (65.3)	
From primary to revision ACLR			35.0 ± 28.2		
From primary to contralateral ACLR					44.1 ± 31.7

*Note*: Data are reported as number (percentage) unless otherwise indicated. Cumulative prevalence: ^a^ 24.5%; ^b^ 26.5%; ^c^ 29.7%.

Abbreviations: ACLR, anterior cruciate ligament reconstruction; BPTB, bone‐patellar tendon‐bone; HT, hamstring tendon; LM, lateral meniscus; MM, medial meniscus; QT, quadriceps tendon; SD, standard deviation.

### Subjective knee function

Preoperatively, there were significant but nonclinically relevant differences in favour of the revision ACLR group for KOOS Symptoms, Pain, ADL and Sport/Rec subscale scores compared with the primary ACLR group. Postoperatively, except for the 1‐year Symptoms and ADL subscales (for which there were no significant differences), the revision ACLR group reported significantly lower scores on all KOOS subscales than the primary ACLR group, with clinically relevant differences (>8–10 points) for the 1‐, 2‐ and 5‐year Sport/Rec and QOL subscales. The bilateral ACLR group reported significantly, but not clinically relevant, inferior scores on the 1‐year Symptoms and QOL subscales and the 5‐year Sport/Rec and QOL subscales compared to the primary ACLR group (Table 2).

**Table 2:** Preoperative and postoperative KOOS comparison.

	Isolated primary ACLR	Revision ACLR^a^	Bilateral ACLR^a^	Isolated primary vs. revision ACLR *p* value	Isolated primary vs. bilateral ACLR *p* value
Symptoms					
Preoperatively	75.9 ± 17.7 (5825)	78.3 ± 17.9 (321)	75.1 ± 17.7 (371)	0.01	n.s.
1 year	80.6 ± 16.5 (4482)	79.6 ± 17.4 (242)	77.7 ± 17.0 (282)	n.s.	0.004
2 year	82.4 ± 16.0 (3244)	77.0 ± 17.6 (159)	81.0 ± 17.0 (205)	<0.001	n.s.
5 year	84.6 ± 15.8 (2652)	80.2 ± 17.5 (139)	82.4 ± 16.1 (183)	0.004	n.s.
Pain					
Preoperatively	79.8 ± 16.2 (5824)	82.5 ± 16.2 (321)	79.9 ± 15.7 (371)	0.003	n.s.
1 year	87.8 ± 12.9 (4481)	85.2 ± 15.3 (242)	87.2 ± 12.3 (282)	0.01	n.s.
2 year	88.5 ± 13.6 (3244)	83.4 ± 16.8 (159)	87.4 ± 13.6 (204)	<0.001	n.s.
5 year	89.7 ± 13.4 (2652)	86.7 ± 14.1 (139)	88.0 ± 13.0 (183)	0.01	n.s.
ADL					
Preoperatively	87.5 ± 14.9 (5822)	90.2 ± 13.5 (321)	88.5 ± 13.4 (371)	0.001	n.s.
1 year	94.1 ± 10.1 (4482)	93.0 ± 11.4 (242)	93.8 ± 9.4 (282)	n.s.	n.s.
2 year	94.0 ± 10.9 (3244)	91.1 ± 14.1 (159)	93.6 ± 9.5 (204)	0.001	n.s.
5 year	94.5 ± 10.5 (2652)	92.3 ± 13.2 (139)	94.1 ± 9.5 (183)	0.02	n.s.
Sport/Rec					
Preoperatively	49.1 ± 29.3 (5813)	53.5 ± 30.1 (320)	48.3 ± 29.9 (370)	0.01	n.s.
1 year	70.8 ± 24.6 (4482)	61.5 ± 26.9 (242)	68.8 ± 24.8 (282)	<0.001	n.s.
2 year	73.3 ± 24.3 (3244)	60.2 ± 28.7 (159)	70.5 ± 25.0 (204)	<0.001	n.s.
5 year	75.4 ± 24.8 (2650)	65.1 ± 27.0 (139)	70.6 ± 26.2 (183)	<0.001	0.01
QOL					
Preoperatively	39.3 ± 24.2 (5819)	40.8 ± 26.4 (321)	38.6 ± 24.4 (371)	n.s.	n.s.
1 year	63.0 ± 22.8 (4482)	54.2 ± 24.4 (242)	59.4 ± 23.4 (282)	<0.001	0.01
2 year	67.4 ± 22.5 (3242)	52.7 ± 24.7 (159)	64.8 ± 23.8 (204)	<0.001	n.s.
5 year	71.6 ± 22.8 (2649)	57.4 ± 22.9 (139)	67.1 ± 23.4 (183)	<0.001	0.009

*Note*: Data are reported as mean ± SD (*n*).

Abbreviations: ACLR, anterior cruciate ligament reconstruction; ADL, Activities of Daily Living; KOOS, Knee Injury and Osteoarthritis Outcome Score; QOL, Quality of Life.

^a^ Preoperative and postoperative KOOS scores for the revision and bilateral ACLR groups are preoperative, 1, 2 and 5 years after the last ACLR (revision ACLR for the revision ACLR group and contralateral ACLR for the bilateral ACLR group).

## DISCUSSION

The most important finding of the present study was that the revision ACLR group reported significantly lower scores on all KOOS subscales (except the 1‐year Symptoms and ADL subscales) than the primary ACLR group, with clinically relevant differences (>8–10 points) [29, 31] for the 1‐, 2‐ and 5‐year Sport/Rec and QOL subscales, which are considered the most sensitive subscales for measuring changes in knee function [30]. In contrast, the bilateral ACLR group reported significant but not clinically relevant (<8–10 points) inferior scores only in the 1‐year Symptoms and QOL subscales and the 5‐year Sport/Rec and QOL subscales compared with the primary ACLR group. Preoperatively, KOOS Symptoms, Pain, ADL and Sport/Rec subscale scores were significantly better in the revision ACLR group than in the primary ACLR group, though the differences were not clinically relevant.

Several studies have investigated the risk factors for contralateral ACL injury and reconstruction [2, 5, 12, 32, 38]. However, only a few studies have compared subjective knee function between primary and bilateral ACLR, yielding conflicting results. Fältström et al. [11] found inferior knee function and QOL in patients with bilateral ACL injuries compared to those in patients with unilateral ACLR. However, the study population was small and heterogeneous. Some patients suffered from ACL graft rupture, whereas others underwent revision ACLR. Moreover, not all patients in the bilateral ACL injury group underwent reconstruction. In a recent matched‐group analysis with a minimum 5‐year follow‐up, Koca et al. [21] found inferior subjective knee function in patients who underwent bilateral ACLR compared to patients who underwent unilateral ACLR. In contrast, Goddard et al. [17] and Cristiani et al. [10] found no differences in subjective knee function between bilateral and unilateral ACLR. Our findings are consistent with those of the latter studies. In the present study, the bilateral ACLR group reported inferior scores only in the 1‐year Symptoms and QOL subscales and the 5‐year Sport/Rec and QOL subscales compared to the primary ACLR group. However, these differences were minor and well below those considered clinically relevant (8–10 points) [29, 31]. Statistical significance was achieved owing to the large sample size.

Although this was a large cohort study comparing subjective knee function between primary and revision ACLR, our findings are in line with those of previous studies with smaller samples which found inferior subjective knee function after revision ACLR compared to primary ACLR [1, 3, 16, 20, 22, 37]. Cristiani et al. [3] reported inferior postoperative KOOS scores after revision ACLR compared with primary ACLR at the 1‐year follow‐up in the same cohort of patients. Similarly, Lind et al. [26] found inferior KOOS scores with revision ACLR compared with primary ACLR at the 2‐ to 9‐year follow‐up. There are several reasons for worse postoperative subjective knee function after revision ACLR. The patients had two severe knee injuries (ACL injury and ACL graft tear) and underwent two surgeries with multiple graft harvesting [3, 18, 37]. Moreover, as in the present study, patients undergoing revision ACLR generally have a higher rate of cartilage injuries and meniscal resections than those undergoing primary ACLR. Finally, another reason for the inferior subjective knee function after revision ACLR compared with primary ACLR in this study might be the much higher rate of BPTB ACLRs in revision surgery than in primary surgery (74.3% vs. 5.7%, respectively). BPTB grafts have been associated with inferior KOOS subscale scores after ACLR [6, 9].

Interestingly, despite not being clinically relevant (<8–10‐point difference) [29, 31], preoperative KOOS scores were higher in the Symptoms, Pain, ADL and Sport/Rec subscales for revision ACLR than for primary ACLR. Similar findings have been reported previously [3, 37]. It has been hypothesised that ACL reinjury has an inferior impact on the perceived life situation compared to the first ACL tear [3].

Regarding the patients' characteristics of the present study, it is interesting to note that the mean age at primary ACLR was lower for the revision and bilateral ACLR groups compared with the isolated primary ACLR group (23.1 ± 7.8 and 24.5 ± 9.1 years vs. 29.2 ± 10.9 years). This is in line with previous studies reporting that a younger age is associated with revision and contralateral ACLR [4, 5]. The proportion of females was higher in the bilateral ACLR group than in the revision ACLR and isolated primary ACLR groups (51.0% vs. 44.9% and 45.4%, respectively). Shelbourne et al. [32] reported that females have a higher risk of contralateral ACL injury than men. Finally, the time from injury to primary ACLR was shorter for the revision and bilateral ACLR groups (9.3 ± 16.7 and 13.7 ± 30.3 months, respectively) compared with the isolated primary ACLR group (21.2 ± 41.9 months). Previous studies have reported that a shorter time from injury to primary ACLR is associated with increased odds of revision and contralateral ACLR [4, 5].

Revision and contralateral ACLR are traumatic events that require repeated long rehabilitation processes. Patients require thorough counselling regarding their expectations postoperatively. The findings presented in this study are helpful for clinicians in clinical practice to inform patients about their expected knee function after a second surgery (revision or contralateral ACLR) compared to the primary surgery. Patients should be aware that revision ACLR, but not bilateral ACLR, is associated with clinically relevant inferior subjective knee function compared to primary ACLR.

The main strength of this study was the large cohort (6831 patients), which compared subjective knee function in patients with revision or bilateral ACLR and primary ACLR. All surgeries were performed at the same institution using a standardised surgical technique, and rehabilitation was also standardised. The follow‐up period was mid‐term (5 years). Finally, no matching or statistical corrections for patient demographics, associated injuries or graft choice between the groups were made; therefore, the results of this study mimic what occurs in the clinical setting.

The main limitations were the 2‐ and 5‐year losses to follow‐up. Loss to follow‐up is a well‐known issue in studies involving large registries [6, 8, 23]. However, we were able to collect 2‐ and 5‐year KOOS scores for 3608 and 2974 patients, respectively, and these samples give high statistical power and generalisability. Another limitation was the lack of data on the return to sports. Unfortunately, our database does not contain this information. Therefore, it was not possible to compare return to sports rates between the study groups.

## CONCLUSIONS

Revision ACLR, but not bilateral ACLR, was associated with clinically relevant inferior subjective knee function compared to primary ACLR. It is important to counsel patients regarding their future subjective knee function after repeated ACLR. Inferior subjective results should be expected after revision ACLR, but not after bilateral ACLR, compared to primary ACLR.

## AUTHOR CONTRIBUTIONS

**Riccardo Cristiani**: Conceptualisation; data curation; methodology; writing. **Eric Hamrin Senorski**, **Camilo P. Helito** and **Kristian Samuelsson**: Methodology; critical review of the manuscript. **Anders Stålman**: Conceptualisation; methodology; critical review of the manuscript. All authors have given final approval of the version to be published.

## CONFLICT OF INTEREST STATEMENT

The authors declare no conflict of interest.

## ETHICS STATEMENT

Ethical approval for this study was obtained from the regional ethics committee, Karolinska Institutet, Diarenumber 2016/1613‐31/32. No consent to participate is requested for registry studies.

## Data Availability

The data that support the findings of this study are available from the corresponding author [R. C.], upon reasonable request.
